# Plasmid-powered evolutionary transitions

**DOI:** 10.7554/eLife.33383

**Published:** 2017-12-12

**Authors:** Ryan A Melnyk, Cara H Haney

**Affiliations:** 1 Department of Microbiology and ImmunologyUniversity of British ColumbiaVancouverCanada; 2 Michael Smith LaboratoriesUniversity of British ColumbiaVancouverCanada

**Keywords:** mutualism, virulence, evolutionary transition, horizontal gene transfer, *Rhodococcus*, plant pathology, Other

## Abstract

The acquisition of a virulence plasmid is sufficient to turn a beneficial strain of *Rhodococcus* bacteria into a pathogen.

**Related research article** Savory EA, Fuller SL, Weisberg AJ, Thomas WJ, Gordon MI, Stevens DM, Creason AL, Belcher MS, Serdani M, Wiseman MS, Grünwald NJ, Putnam ML, Chang JH. 2017. Evolutionary transitions between beneficial and phytopathogenic *Rhodococcus* challenge disease management. *eLife* **6**:e30925. doi: 10.7554/eLife.30925

Many bacteria are closely associated with eukaryotic hosts. This relationship can be mutually beneficial, commensal (that is, it benefits one party without affecting the other), or pathogenic. Moreover, and perhaps surprisingly, there are many examples of pathogenic bacteria that are close relatives of mutualistic or commensal bacteria (Wood et al., 2001). For example, while most strains of *E. coli* are commensal, pathogenic strains – such as those that cause food poisoning – emerged from commensal ancestors (Pupo et al., 2000).

Historically, the bacterial genus *Rhodococcus* has been synonymous with pathogenesis because most described strains could cause a plant disease called leafy gall and stunt the growth of their plant hosts (Stes et al., 2011). Recently, it was found that some *Rhodococcus* strains are benign, and some are constituents of a healthy microbiome in plants (Bai et al., 2015; Hong et al., 2016). Now, in eLife, Jeff Chang of Oregon State University and co-workers – including Elizabeth Savory, Skylar Fuller and Alexandra Weisberg as joint first authors – report using a collection of *Rhodococcus* strains to study the molecular mechanisms that underlie distinct bacterial lifestyles (Savory et al., 2017).

Savory *et al*. isolated 60 strains of *Rhodococcus* bacteria from healthy and diseased plants from 16 nurseries over a period of several years and then sequenced their genomes and plasmids (which usually contain the virulence genes which make *Rhodococcus* pathogenic). The researchers compared pathogenic and commensal strains and used single nucleotide polymorphisms to reconstruct evolutionary histories within the *Rhodococcus* genus. They found relatively little correlation between the genome sequences of a strain and the DNA sequences in the plasmids. In particular, they found that isolates with similar genomes could have plasmids with radically different virulence genes, while plasmids with similar virulence genes could be found in strains with very different genomes. This suggests that the emergence of pathogenic strains may be driven by the transfer of plasmids between strains that are relatively evolutionarily distant from each other.

The researchers identified *Rhodococcus* isolates that lack a virulence plasmid but are close relatives of isolates that possess a plasmid. They showed that *Rhodococccus* isolates that lack a virulence plasmid can colonize *Nicotiana benthamiana*, a model plant that is susceptible to *Rhodococcus* infection. However, rather than causing disease, the strains lacking a virulence plasmid promoted the development of lateral roots and root hairs (Figure 1). This indicates that beneficial and pathogenic *Rhodococcus* are closely related, which led Savory *et al*. to hypothesize that the commensal and pathogenic *Rhodococcus* may evolve by the acquisition or loss of a virulence plasmid.

**Figure 1.**
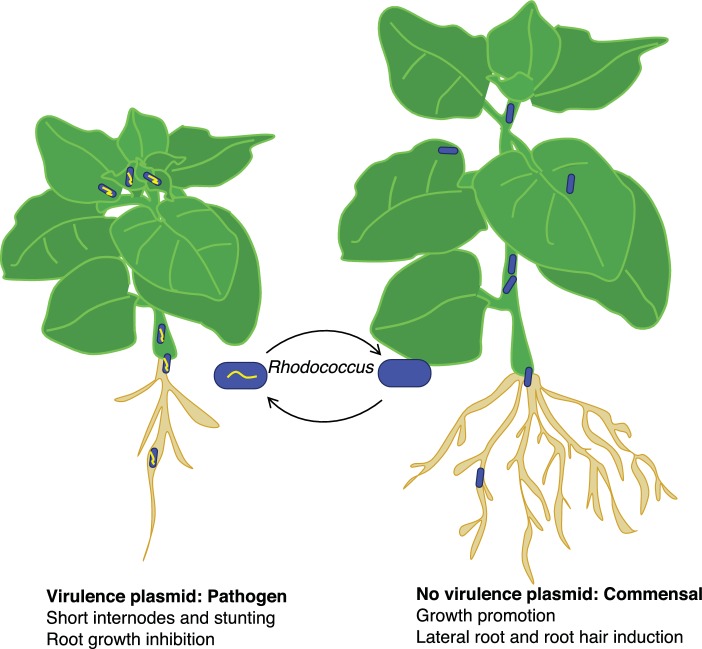
The good and bad sides of *Rhodococcus.* The acquisition of a virulence plasmid (yellow) can transform benign commensal strains of *Rhodococcus* (blue) into pathogenic strains (blue and yellow) that stunt the growth of the plant and its roots (left) compared with a healthy plant (right). However, this process is reversible, and the loss of the virulence plasmid results in the bacteria becoming commensal again.

Savory *et al*. went on to experimentally demonstrate that pathogenic *Rhodococcus* strains can evolve from commensals. They showed that the introduction of a virulence plasmid from a pathogenic isolate into a beneficial strain was sufficient to cause disease in *N. benthamiana*. They also showed that the transition from commensal to pathogen is reversible: plasmid loss causes a virulent strain to become beneficial. By combining experimental data with genomic evidence, Savory *et al*. conclusively demonstrate that a readily transmissible plasmid is necessary and sufficient to reversibly transform beneficial *Rhodococcus* strains into pathogens.

Recently, it has been alleged that various strains of *Rhodococcus* are the cause of “pistachio bushy top syndrome” (PBTS), which has affected millions of pistachio trees in the United States and resulted in substantial economic losses (Stamler et al., 2015). However, rapid evolutionary changes present a challenge in identifying the pathogenic agents responsible for PBTS. Savory *et al*. tested the virulence of two *Rhodococcus* strains purported to be the causative agents of PBTS on several different hosts: they found that both isolates were not pathogenic. Moreover, they report that they were unable to amplify *Rhodococcus* virulence genes by PCR. This, combined with the fact that the published genomes of these strains do not contain virulence loci (Stamler et al., 2016), suggests that the plasmid was lost during isolation or that the disease was misdiagnosed.

The ambiguity of the cause of PBTS underscores the need for multiple independent methods that can verify the identity of an emerging plant pathogen, particularly when it has benign close relatives. To this end, Savory *et al*. developed an alternative method of identifying virulent strains of *Rhodococcus* based on recombinase polymerase amplification, and showed that this test could robustly discriminate between pathogenic and beneficial strains by targeting conserved loci in the virulence plasmid.

The work of Savory *et al*. provides insights into the evolutionary history of plant pathogens and mutualists. In particular, the coincidence of virulence and mutualism within the *Rhodococcus* clade suggests that both strategies are adaptive under certain conditions: sustained positive selection for one lifestyle would be unlikely to result in closely related strains with dramatically different effects on plant health. Building on this work may illuminate the evolutionary mechanisms that drive transitions from beneficial to pathogenic lifestyles in bacteria. This in turn could have profound implications for the management of emerging agricultural diseases and the evolution of plant-microbe interactions.
